# Enhancing Haloarene Coupling Reaction Efficiency on an Oxide Surface by Metal Atom Addition

**DOI:** 10.1021/acs.nanolett.3c04111

**Published:** 2024-02-05

**Authors:** Mikel Abadia, Ignacio Piquero-Zulaica, Jens Brede, Alberto Verdini, Luca Floreano, Johannes V. Barth, Jorge Lobo-Checa, Martina Corso, Celia Rogero

**Affiliations:** † Centro de Física de Materiales (CSIC-UPV/EHU), Materials Physics Center MPC, Paseo Manuel de Lardizabal 5, E-20018 San Sebastián, Spain; ‡ Donostia International Physics Center (DIPC), Paseo Manuel de Lardizabal 4, E-20018 Donostia-San Sebastián, Spain; § Physics Department E20, Technical University of Munich (TUM), 85748 Garching, Germany; ∥ CNR-IOM, Instituto Officina dei Materiali Laboratorio TASC, 34149 Trieste, Italy; ⊥ Instituto de Nanociencia y Materiales de Aragón (INMA), CSIC-Universidad de Zaragoza, 50009 Zaragoza, Spain; # Departamento de Física de la Materia Condensada, Universidad de Zaragoza, 50009 Zaragoza, Spain

**Keywords:** Oxides, On-surface synthesis, Single-atom catalyst, Ullmann coupling, X-ray photoelectron spectroscopy

## Abstract

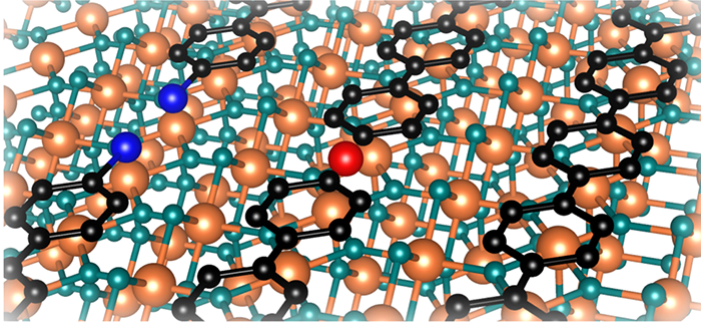

The bottom-up synthesis of carbon-based nanomaterials directly on semiconductor surfaces allows for the decoupling of their electronic and magnetic properties from the substrates. However, the typically reduced reactivity of such nonmetallic surfaces adversely affects the course of these reactions. Here, we achieve a high polymerization yield of halogenated polyphenyl molecular building blocks on the semiconducting TiO_2_(110) surface via concomitant surface decoration with cobalt atoms, which catalyze the Ullmann coupling reaction. Specifically, cobalt atoms trigger the debromination of 4,4″-dibromo-*p*-terphenyl molecules on TiO_2_(110) and mediate the formation of an intermediate organometallic phase already at room temperature (RT). As the debromination temperature is drastically reduced, homocoupling and polymerization readily proceed, preventing presursor desorption from the substrate and entailing a drastic increase of the poly-*para*-phenylene polymerization yield. The general efficacy of this mechanism is shown with an iodinated terphenyl derivative, which exhibits similar dehalogenation and reaction yield.

Throughout the past decade, on-surface chemistry has proven to be an extraordinary tool for building sp^2^ bond-based carbon nanostructures with unprecedented atomic precision.^1−4^ Motivated by the fact that such carbon structures, which are hard to synthesize by common wet chemistry methods, have prominent electronic properties deserving implementation into electronic devices, the interest of exploring surface-induced molecular reactions has grown exponentially.^5−9^

The Ullmann coupling reaction, in conjunction with a subsequent cyclodehydrogenation step, is recognized as the most promising pathway toward designing carbon-based nanostructures for molecular electronics on surfaces.^2,10−12^ However, bringing such materials into nanodevices remains a challenge, as the surface-assisted Ullmann reaction is mostly employed in ultrahigh vacuum (UHV) conditions on single-crystal noble metal substrates (gold,^13^ silver,^14^ and copper)^15^ given their catalytic activity. Nevertheless, these metal-adsorbed nanostructures present limitations since they remain electronically coupled to the underlying catalyzing substrate. Thus, the decoupling of the synthesized nanostructures must be achieved afterward for their implementation into devices,^16^ which is currently performed by cumbersome postgrowth transfer methods.

The on-surface synthesis of molecular nanostructures directly on semiconductors and insulators would allow one to overcome such fundamental problems. To date, successful reports of this approach are very scarce given the limited catalyzing activity of these substrates.^17−20^ Among these, we have demonstrated in previous works^21,22^ that the on-surface Ullmann coupling reaction can be directly catalyzed by semiconductor surfaces such as TiO_2_(110), exploiting undercoordinated subsurface interstitial Ti atoms. Recently, Zuzak et al.^23^ followed the same procedure to synthesize different types of nanographenes on a reduced TiO_2_(110) sample. Before them, Kolmer et al.^24,25^ also demonstrated the on-surface synthesis of 7-AGNRs using a de novo synthesized fluorinated precursor on the TiO_2_(011) surface.^26,27^ Other catalysts, such as Cu or Pd, have also been used to induce Ullmann-like reactions on a hexagonal boron nitride (h-BN) decoupling layer grown on a Ni(111) substrate.^18^

The extremely low yields of the Ullmann coupling reaction on semiconductor surfaces are related to two fundamental reasons: (i) the lack of surface catalytic capability to induce molecular dehalogenation and (ii) the relatively high temperatures that are required for dehalogenation often come close or overlap with the temperature threshold for molecular desorption or even decomposition. In the present work, we demonstrate a new pathway to overcome the above-mentioned limitations and significantly improve the Ullmann-like coupling reaction yield of halogenated molecules on the TiO_2_(110) surface. By adding minute amounts of cobalt atoms in the initial stages of the reaction, a dramatic improvement of the polymerization yield of 4,4″-dibromo-*p*-terphenyl (DBTP) molecules into poly-*para*-phenylene (PPP) polymers by almost 300% is observed. The generality of the mechanism is demonstrated by obtaining a similar efficiency for iodinated terphenyl derivative (DITP) molecules. We unambiguously show this by means of synchrotron-based X-ray photoemission spectroscopy (XPS), lab-based angle-resolved photoemission spectroscopy (ARPES), low-energy electron diffraction (LEED), and room-temperature (RT) scanning tunneling microscopy (RT-STM) techniques.

The measurements were obtained as follows: on the one hand, a sample consisting of a single layer of DBTP deposited on the TiO_2_(110) surface was used for control and comparison purposes (from here on, this is termed the “control sample”). The molecular adsorption was self-limited to a single layer.^15^ On the other hand, a second sample was similarly prepared with a single layer of DBTP deposited on the TiO_2_(110) surface, but here, in a second step, cobalt atoms were thermally evaporated onto the sample kept at room temperature (RT) (from here on, this is termed the “cobalt sample”). The temperature evolution of the surface reactants was then systematically measured for both samples in equivalent conditions, and the differences within the reaction pathway were determined by photoemission techniques.

In order to limit the catalytic contribution of subsurface Ti interstitial atoms,^28,29^ a nearly stoichiometric TiO_2_(110) surface was used throughout this work (see Figure S1 in the Supporting Information (SI)), whereas purposely reduced samples were used in previous studies.^21,23^

The molecular structure of DBTP and the atomic structure of TiO_2_(110) are shown in panels a and b of Figure 1, respectively. Panels c and d of Figure 1 show the Br 3d and C 1s core level (CL) XPS spectra obtained at RT for the control (in black) and cobalt (in red) samples. The C 1s CL peak of the control sample in Figure 1d was deconvoluted with three components, in agreement with previous works,^21,22^ i.e., two halogen-bonded carbons at 285.8 eV (C–Br), four carbons in the *para* position of the benzene rings at 284.9 eV (C–C), and 12 carbons of the molecular backbone with the lowest binding energy (BE) at 284.7 eV (C–H). The BE of the spin–orbit coupled Br 3d double peaks shown in Figure 1c indicate that the DBTP molecules remained halogenated after deposition onto the TiO_2_(110) surface^22^ (see Figure 1b).

**Figure 1:**
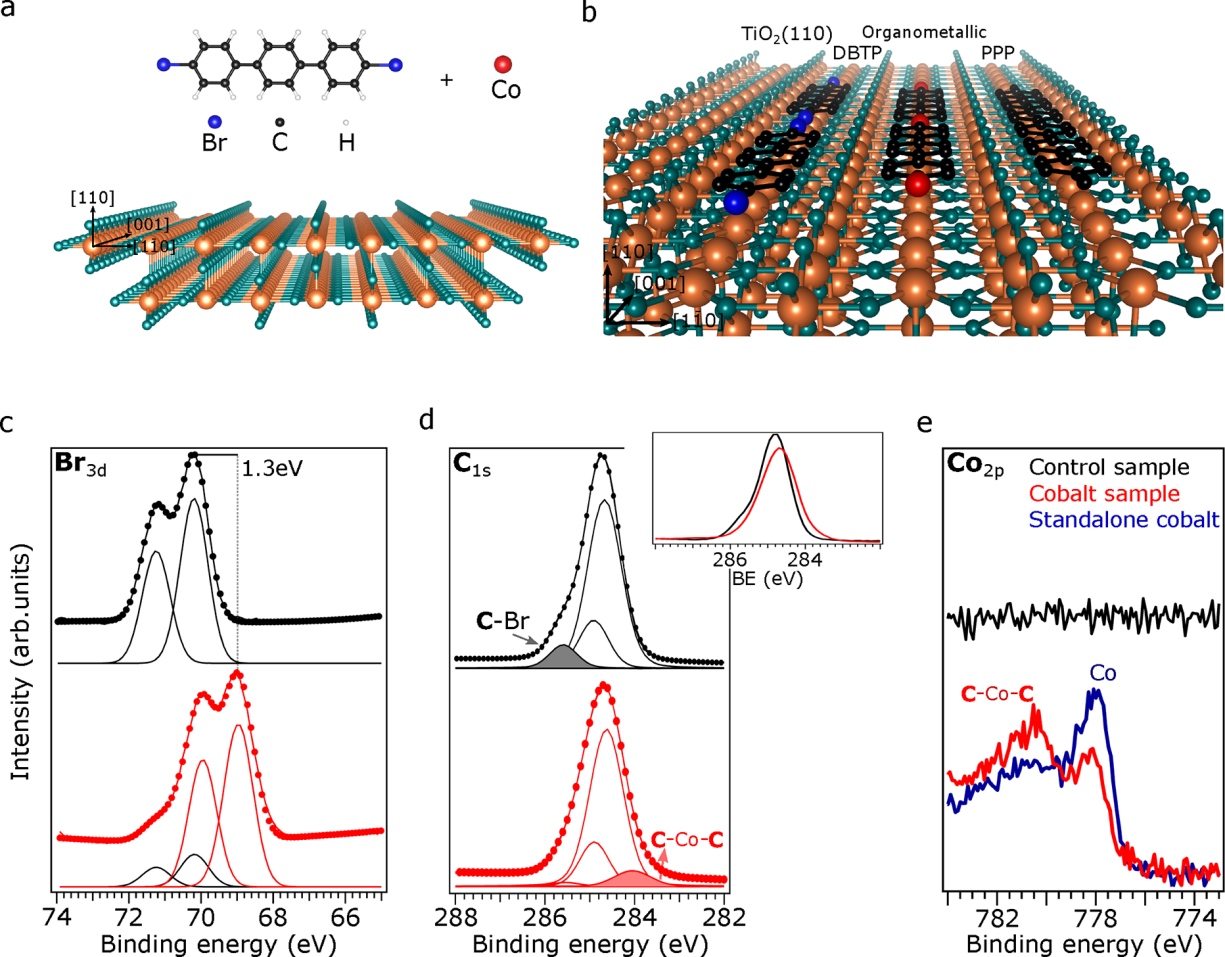
(a) Ullmann-like process constituents, i.e., DBTP as the molecular precursor, cobalt as the external catalyst, and the template substrate TiO_2_(110). (b) DBTP adsorption on the TiO_2_(110) trenches is shown, as well as the intermediate organometallic phase and the final PPP reaction product. XPS measurements for DBTP on the control (black spectra) and cobalt samples (red spectra) for (c) Br 3d, (d) C 1s, and (e) Co 2p CLs. In the last panel, the Co 2p CL spectrum of cobalt atoms as deposited on the clean TiO_2_(110) surface is included for direct comparison (blue spectrum). The inset in (d) compares the C 1s peak shape of the control and cobalt samples. Measurements were obtained with the samples at RT (see the Methods section in the SI).

We found significant changes in the cobalt sample for both the Br 3d and C 1s peaks (red spectra in panels c and d of Figure 1, respectively). The most striking one was the presence of an additional component of the Br 3d CL, shifted by 1.3 eV to a lower BE (from 70.6 to 69.3 eV). Similarly to noble metal surfaces,^30^ the observed shift was ascribed to the debromination of the DBTP molecules. In contrast, the debromination of DBPT molecules did not occur before 450 K in the control sample, as previously reported^21,22^ and evidenced in Figure 2d.

**Figure 2:**
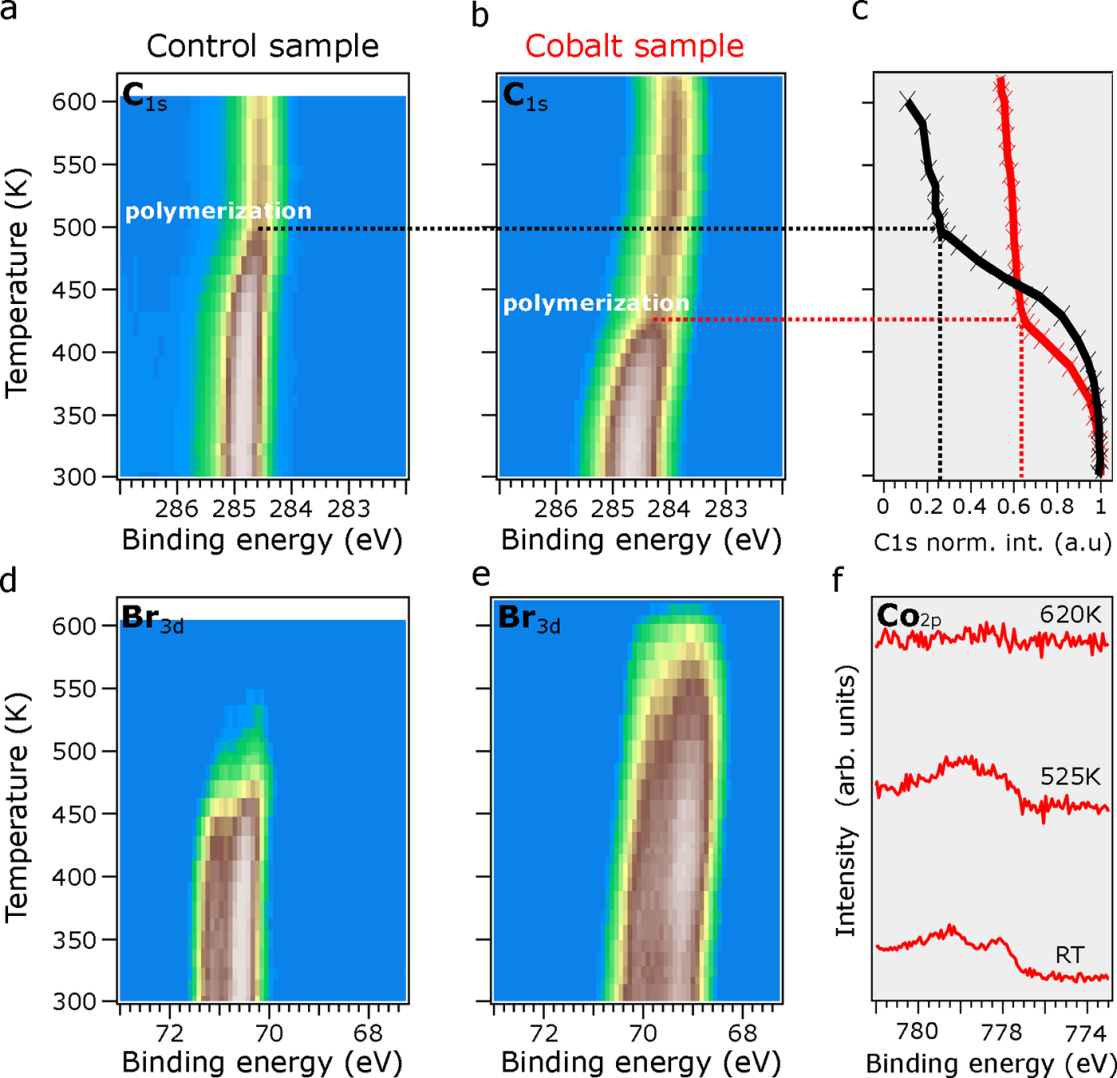
Comparison of the TD-XPS measurements of the C 1s and Br 3d CLs of DBTP in the (a, d) control and (b, e) cobalt samples. Fast XPS acquisition was completed while heating the samples from 300 K to 620 K with a constant linear ramp of 7.5 K/min. In (c), the normalized integrated area of C 1s CL is plotted against temperature for both samples. (f) The Co 2p CL peak of the cobalt sample at three selected temperatures: RT, 525 K, and 620 K.

Consistent differences also emerged when comparing the C 1s spectra of both samples. The inset top image in Figure 1d shows a decrease in the signal intensity at the high BE side of the red spectrum, accompanied by an intensity increase on the low BE side. Interestingly, the overall C 1s CL integrated intensity area remained unchanged between both spectra, indicating that no molecular desorption occurred upon the addition of cobalt atoms. The deconvolution of the peak, as seen in the red spectrum in Figure 1d, further accentuates the observed differences. On the one hand, the high BE peak at 285.5 eV (C–Br) practically disappears, which has recurrently been assigned to the debromination of DBTP molecules.^5,14,15,21,22^ On the other hand, the emergence of a new peak component at 284 eV points to the formation of an organometallic intermediate phase (C–Co–C bonding motif), where cobalt atoms link the debrominated radical carbon atoms of adjacent molecules to stabilize their charge, as illustrated in Figure 1b. We note that, while similar organometallic phases or intermediates have frequently been reported on metallic Cu or Ag surfaces,^15^ this is, to the best of our knowledge, the first observation of such a phase on a semiconducting surface.

The formation of the organometallic phase is also evident in the Co 2p CL spectra in Figure 1e. The blue spectrum represents the adsorption of cobalt on the clean TiO_2_(110) surface, with a pronounced peak at 778 eV corresponding to its metallic nature. In the presence of the DBTP monolayer, however, the peak shifts toward a higher BE, proving the strong chemical interaction of the precursor molecules with cobalt atoms.

We further followed the polymerization reaction on both the reference sample and the cobalt sample by temperature-dependent XPS (TD-XPS) measurements, as shown in Figure 2. Here, both samples were annealed from 300 K (RT) to 620 K while the C 1s and Br 3d CLs of DBTP were monitored. Unfortunately, the minute amounts of Co prevented us from visualizing such an evolution within a similar time frame; therefore, we select three temperatures to acquire Co 2p spectra with the necessary statistics. In this way, three major spectroscopic fingerprints were singled out after comparing both samples: the molecular debromination temperature difference (already evident at RT for the cobalt sample), the identification of the homocoupling temperature, and the final C 1s CL signal intensity, directly correlated with the reaction efficiency.

The temperature evolution of the C 1s and Br 3d CLs of the control sample (panels a and d of Figure 2, respectively) have been analyzed elsewhere,^21,22^ and the results are summarized as follows: at 475 K, the Br 3d CL signal intensity dramatically drops due to DBTP debromination and the subsequent desorption of the bromine atoms from the surface. At this temperature, the intensity of the C 1s CL also drops to roughly a quarter of its initial value (see the dashed black line in the intensity profile shown in Figure 2c). This demonstrates that a significant number of DBTP molecules desorb from the surface before polymerizing. The remaining C 1s signal intensity arises from the successfully polymerized molecules, as explained below.

The observed low reaction efficiency on the control sample (≈25%) is ascribed to two main factors: the first is that intrinsically, only very few sites exist that can catalytically activate molecular debromination on the reduced TiO_2_(110) surface. These are mostly surface defects with an excess of accessible charge, often produced by standard UHV sample preparation procedures. In the present case, the presence of such defects was deliberately minimized to preserve the pristine properties of the TiO_2_(110) surface (nonreduced). The second reason is that the thermally activated outward diffusion of Ti interstitials^31^ takes place in the same temperature range as DBTP debromination and molecular desorption.

In the following, we show how lowering the debromination temperature (by 150 K) dramatically affects the Ullmann-like reaction yield on the TiO_2_(110) surface. The TD-XPS results of the cobalt sample presented clear differences in both core level lines. The decrease in intensity of the C 1s CL signal (see Figure 2b), already occurred at 420 K, i.e., there was a 55 K reduction compared to the control sample. Remarkably, the total signal intensity in Figure 2c is almost 3 times higher compared to that of the control sample (about 60% of the RT signal), which observation proved that molecular desorption was considerably reduced. Since the cobalt atoms already activated the debromination of the DBTP molecules at RT, most of the intermolecular homocoupling occurred well below their surface desorption temperature.

The temperature evolution of the Br 3d CL was also considerably different on the cobalt sample. The surface desorption of bromine atoms started at 550 K, i.e., 75 K higher than on the control sample. We correlate this higher desorption temperature to the presence of cobalt atoms on the surface leading to a Br–Co complex, as evidenced by the change in the Co 2p line shape in Figure 2f. The high-resolution XPS spectra of Co 2p CL showed the presence of cobalt atoms on TiO_2_(110) at RT and 525 K but not at 620 K, which is in agreement with the changes observed in the C 1s and Br 3d CLs. We interpret the temperature evolution of the Br 3d and Co 2p CLs as the formation of a Br–Co complex, which desorbs at around 600 K. Note that the C 1s signature remained constant at this temperature, proving that the homocoupling reaction had taken place and the polymers remained on the TiO_2_(110) surface (see Figure 1b).

So far, these core-level spectroscopic characterizations give strong support of the cobalt-induced molecular dehalogenation at RT and the subsequent organometallic bonding and homocoupling of the dehalogenated species at 420 K. The eventual formation of PPP chains was further confirmed by LEED images obtained at several steps of the reaction, as well as by RT-STM imaging after the 450 K annealing step (see Figures S2 and S3).

The ultimate proof of the successful reaction is the delocalization of the atomic carbon p_*z*_ orbitals along the entire polymer. Experimentally, such a delocalization manifests in a highly dispersive π-band,^15,32^ which can be directly measured by ARPES.

In Figure 3, ARPES intensity maps of the control and cobalt samples are presented. The control sample spectrum was measured after annealing to 475 K, and the cobalt sample spectrum was measured after annealing to 425 K. The RT spectra of both samples are shown in the SI, Figure S4.

**Figure 3:**
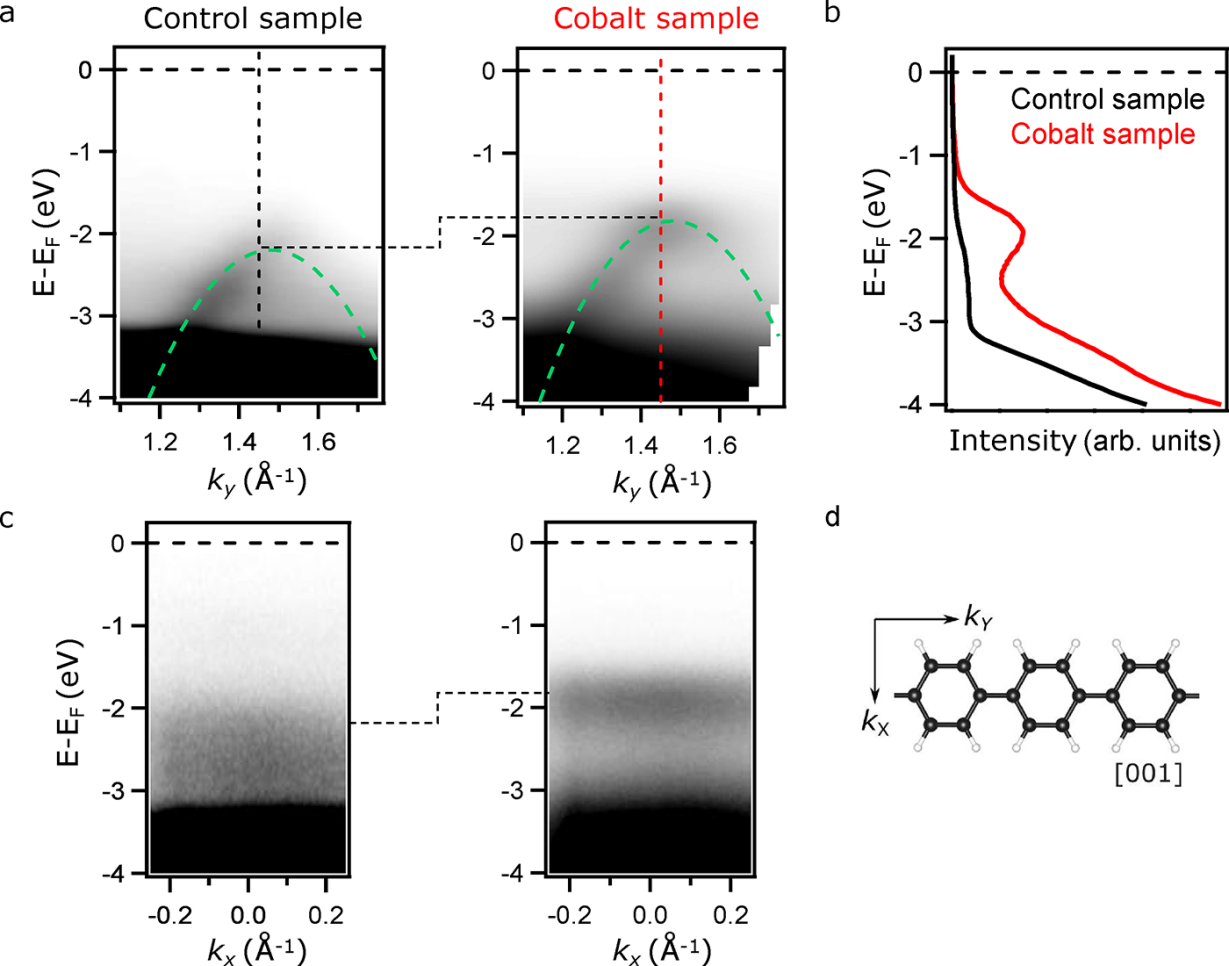
Band structure measured by ARPES of PPP polymers grown on TiO_2_(110) for both the control and cobalt samples after annealing to 475 K and 425 K, respectively. (a) *E* vs *k*_*y*_ photoemission intensity maps, where the down-dispersive parabolic band along the PPP polymer axis is detected. (b) Comparison of EDC profiles extracted at k_*y*_ = 1.45 Å^–1^ for both samples measured in exactly the same experimental conditions. (c) *E* vs *k*_*x*_ photoemission intensity maps extracted at k_*y*_ = 1.45 Å^–1^ for both samples. (d) Molecular model of PPP, along with the wave vector directions of the photoemission measurements.

The ARPES maps in Figure 3a correspond to the PPP band dispersion parallel to the chains, i.e., along the sample [001] direction (see Figure 3d). In both cases, the photoemission intensity presented a highly dispersive band with its apex located at *k*_*y*_ = 1.45 Å^–1^, in line with the polymer interphenyl distance periodicity.^21,32^ Green dashed lines account for the expected dispersion of the PPP polymeric bands calculated by using an effective mass of m* = 0.2*m*_e_, in agreement with previous work.^15^ Such an effective mass is only possible in polyphenelene covalent polymers, whereas for the organometallic intermediates and nonpolymerized DBTP molecules, discrete nondispersive bands of the molecular orbitals can be found in the ARPES spectra, as shown in the SI, Figures S4 and S5a.

The improvement of the reaction yield (60% versus 25%) was evidenced by the stronger intensity of the cobalt sample compared to that of the control sample, which is highlighted in the energy distribution curves (EDCs) extracted at *k*_*y*_ = 1.45 Å^–1^ (shown in Figure 3b) and in the SI, Figure S5 (second derivative ARPES band structure). Due to the absence of the TiO_2_(110) surface defect state (DS), otherwise located close to −1 eV,^21,33^ the first spectral intensity that emerged in both samples was the valence band (VB) onset located at around −2 eV. In the control sample (black spectrum), the feature was rather weak when compared to the pronounced peak present in the cobalt sample (red spectrum). As already indicated, this intensity difference can be directly ascribed to the signals arising from the number of PPP chains on the surface. Thus, the combination of ARPES and XPS unambiguously shows the benefit of introducing minute amounts of Co for promoting the Ullmann-like process on the TiO_2_(110) surface.

The presence of Co and Br atoms on the surface induces electronic doping in both the organometallic intermediates and the polymer chains (see the SI, Figure S4, for further details). In Figure 3c, ARPES intensity maps of the band structure perpendicular to the top of the PPP band at k_*y*_ = 1.45 Å^–1^ are shown (see Figure 3d). The black line highlights the shift of about 0.4 eV of the top of the molecular π-band toward the Fermi level of the cobalt sample with respect to the control sample. Similarly, a ≈0.6 eV shift was observed at RT after the formation of the organometallic intermediate with the addition of cobalt atoms. Piquero-Zulaica et al.^34^ found such a shift by scanning tunneling spectroscopy (STS) measurements, when zigzag-shaped poly-*meta*-phenylene polymers were in contact with Br atoms in metallic surfaces; the effect has also been theoretically elucidated by Maier et al.^35^ Thus, considering that in the cobalt sample, the Br atoms remained on the TiO_2_(110) surface up until 600 K, we assign the observed band energy shift to the presence of bromine and cobalt atoms surrounding the PPP polymer (see Figure S3). Note that for the control sample, the bromine atoms were almost entirely desorbed at 475 K (Figure 2d).

Finally, we extend this study to the iodine terminated triphenyl sister precursor (DITP). An analogous experimental design to that described above was used, where a control sample (DITP) and a cobalt sample (i.e, cobalt and DITP) were prepared and compared. Then, similarly to what is shown in Figure 2, TD-XPS measurements of the Ullmann-like coupling and polymerization process of DITP upon the presence of Co catalysts on the surface were obtained. The results are summarized in Figure 4.

**Figure 4:**
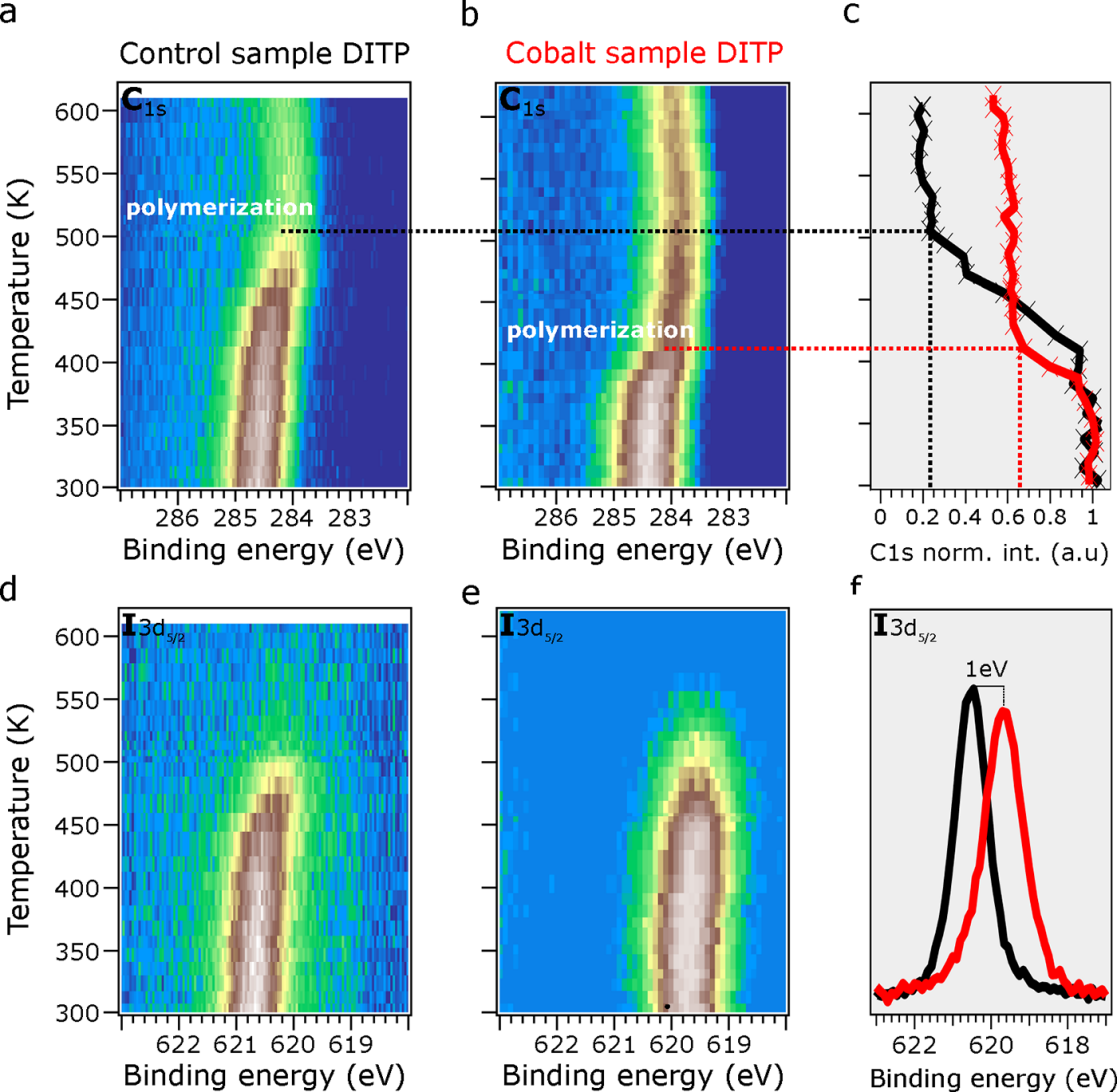
Comparison of TD-XPS measurements of the C 1s and I 3d CLs of DITP in the (a, d) control and (b, e) cobalt samples. The temperature was increased from 300 (RT) to 620 K. In (c), the normalized integrated intensity area of C 1s CL is plotted against temperature for both samples. (f) Room-temperature high-resolution spectra of the I 3d CL for both samples (control sample in black and cobalt sample in red).

Similarly to what happened for DBTP, the dehalogenation of DITP occurred at RT upon the addition of cobalt atoms, as seen in Figure 4f. The 1.0 eV shift of the I 3d CL from the control (black line) to the cobalt (red line) samples indicates the formation of an organometallic (C–Co–C) phase, demonstrating that the cobalt atoms effectively catalyzed the dehalogenation of the halogenated molecular precursors.

The TD-XPS analysis of the DITP system revealed distinct differences compared to the DBTP system. Specifically, the surface desorption temperature of iodine in the presence of cobalt was notably lower than that of bromine. As shown in Figure 4e, iodine atoms desorb from the surface at temperatures near 500 K, whereas bromine atoms remained on the surface until temperatures as high as 600 K, as observed in Figure 2e. This difference can be attributed to the higher thermal stability of cobalt–bromine complexes compared to cobalt–iodine complexes.^36^ The determination of the differences in the cobalt-catalyzed halogen scission temperature on TiO_2_(110) is, however, out of the scope of the present work as TD measurements below RT would be required.

Finally, the polymerization temperature of DITP was identified from the C 1s CL shift. In the control sample (Figure 4a), this shift was observed at 475 K and was accompanied by a significant drop in the signal intensity to 25% of the initial value, similar to DBTP. In the presence of cobalt, the polymerization temperature of DITP dropped to 420 K (Figure 4b), and the final signal intensity was 60% of the initial value. This, again, is consistent with the results obtained for DBTP in Figure 2b and demonstrates that, irrespective of the halogen atom present in the precursor molecules, the Ullmann-like process is dramatically improved by using cobalt atoms as a catalyst on the TiO2(110) surface.

From an overarching point of view, these findings suggest that the substantially improved reaction scenario presents an intriguing example of single-atom catalysis massively enhancing an on-surface synthesis protocol. Remarkably, related phenomena were also encountered in different chemical environments, whereby transformations of molecular moieties and coupling phenomena occurred on metal surfaces decorated with hetero-adatoms enhancing catalytic activity.^37^

In conclusion, the use of cobalt atoms is an effective strategy to catalyze the Ullmann-like coupling of terphenyl derivatives on the TiO_2_(110) surface. The yield of their polymerization reaction is practically tripled, and the polymerization temperature is significantly lowered by 55 K. The improvement in the reaction originates from the strong cobalt–molecule interaction, which promotes the dehalogenation and the formation of an organometallic phase even at RT. This allows for the improvement of the probability of the molecules to polymerize afterward, which contrasts with the previously studied cases in the absence of cobalt, where the molecular dehalogenation temperature practically coincided with their surface desorption temperature. Moreover, we confirmed that the presence of bromine and cobalt atoms in the vicinity of the molecules shifts both the nondispersive molecular orbitals of DBTP and the highly dispersive valence band of the PPP polymer toward the Fermi level. Such electronic doping can be used as a simple way to modulate the band energy of conjugated π-bands on the TiO_2_(110) surface. In short, this study presents a new strategy for implementing the on-surface Ullmann-like reaction with high efficiency in poorly reactive semiconducting or insulating surfaces such as TiO_2_(110), opening a promising avenue for synthesizing graphene-based nanostructures, such as graphene nanoribbons and nanoporous graphene structures, directly on more technologically relevant surfaces.

## Supporting Information Available

Methods section (details of sample growth, measurement parameters) and additional experimental data: nonreduced TiO_2_(110) UV spectra, STM and LEED images of PPP on TiO_2_(110), and ARPES data with second derivative analysis (PDF)
