# Supplementary material for: Enhancing Haloarene Coupling Reaction Efficiency on an Oxide Surface by Metal Atom Addition
Source: Nano Lett. 2024 Feb 5;24(6):1923–30. doi: 10.1021/acs.nanolett.3c04111 (PMC10870764; doi:10.1021/acs.nanolett.3c04111)
Supplement: Supplementary file 1 — nl3c04111_si_001.pdf [file nl3c04111_si_001.pdf]

Supplementary Information:

Enhancing haloarene coupling reaction efficiency on an oxide surface by metal atom addition

Mikel Abadia [*1,2,†], Ignacio Piquero-Zulaica[2,3,†], Jens Brede[,1], Alberto Verdini[4], Luca Floreano[4], Johannes V. Barth[3], Jorge Lobo-Checa[5,6], Martina Corso[1,2] and Celia Rogero [*1,2]

[1] Centro de Física de Materiales (CSIC-UPV/EHU), Materials Physics Center MPC, Paseo Manuel de Lardizabal 5, E-200018 San Sebastián, Spain

[2] Donostia International Physics Center (DIPC), Paseo Manuel de Lardizabal 4, E-20018 Donostia-San Sebastian, Spain

[3] Physics Department E20, Technical University of Munich (TUM), 85748 Garching, Germany

[4] Instituto Officina dei Materiali (CNR-IOM), Laboratorio TASC, Trieste, Italy

[†] M.A. and I.P.-Z. contributed equally to this work.

[5] Instituto de Nanociencia y Materiales de Aragón (INMA), CSIC-Universidad de Zaragoza, Zaragoza, 50009, Spain

[6] Departamento de Física de la Materia Condensada, Universidad de Zaragoza, 50009, Zaragoza, Spain

Methods

Experiments were carried out in UHV systems at a base pressure of 10–10 mbar. We performed X-ray photoemission spectroscopy (XPS) measurements at the ALOISA beamline of the Elettra Synchrotron (Trieste, Italy). Measurements were performed in transverse magnetic polarization (i.e., close to p-polarization) and normal emission geometry, with the sample at a grazing angle of 4°. The photoemission spectra of Br 3d and C 1s (measured using hν = 500 eV, DE□160meV) and of the valence band (at hν = 140 eV, DE□115meV) were calibrated to the BE of Ti 3p at 37.6 ± 0.05 eV. The Ti 2p3/2 core level was measured using hν = 650 eV, DE□260meV. High-resolution spectra were recorded at RT. The temperature-dependent fast-XPS scans of the Br 3d doublet were measured using a temperature ramp speed of 7.5 K/min. We employed faster scanning parameters, including higher pass energy and energy steps per scan, compared to those used for high-resolution spectra. This compromise

in XPS spectral resolution was necessary to ensure adequate signal intensity for *in-situ* detecting temperature-induced spectral changes.

For preparing a nearly stoichiometric $TiO_2(110)$ crystal only two or three sputtering–annealing (Ar+, 1 keV-900K) cycles were performed to obtain a slightly conductive surface but retaining the optical transparency of the crystal. 4,4-Dibromo-p-terphenyl (DBTP) molecules (Sigma-Aldrich, purity higher than ≥88%) were sublimated from a degassed Knudsen cell (molecules degassed several hours/days by heating the source to temperatures slightly below 350 K under UHV conditions) heated to about 375 K to obtain a rate close to 0.33 ML/min. Co was deposited from a rod with commercial e-cell (Focus) at pressures below 6 × 10–9 mbar onto the TiO2(110) crystal held at RT. Flux was calibrated with the fluxmeter included in the evaporator and we deposite around 0.1ML of Co on the sample.

Scanning tunneling microscopy was carried out at RT using an Omicron VT-STM. Image processing was done with the WSxM software[1]. The insulating character of the substrate difficult the acquisition of high quality/high resolution image that mostly present spikes, tip crashes and noise.

Angle-resolved photoemission measurements were performed using a Phoibos 150 SPECS high-resolution hemispherical electron analyzer while the sample was cooled to 150 K. He-Iα (hν = 21.2 eV) radiation was provided by a high-intensity UVS-300 SPECS discharge lamp coupled to a TMM-302 SPECS monochromator. All binding energies given here were referenced to the Fermi level (EF), i.e., BE = 0 = EF.

The model of the atomic structure of the $TiO_2(110)$ surface and the chemical structures of DBTP and DITP are done with the VESTA software[2].

Checking the absence of defects on the $TiO_2(110)$ surface

Ti interstitials atoms are naturally formed in $TiO_2(110)$ sample as a byproduct of oxygen reduction which is stimulated by the standard cleaning and ordering protocols in UHV (ion bombardment and high temperature annealing), as well as by X-ray irradiation. The excess of charge associated with oxygen vacancies (even the buried ones) is redistributed among a few characteristic subsurface lattice Ti atoms[3]. Such atoms present clear spectroscopic fingerprints either in the Ti2*p* CL peak, as a shoulder in the lower binding energy site or in the VB, in the form of a pronounced peak around 0.9eV, normally called the defect state (DS) peak. Both

spectroscopic features are representative of the defectiveness of the sample, i.e. of its stoichiometry, as highlighted in the comparative spectra shown in Figure S1.

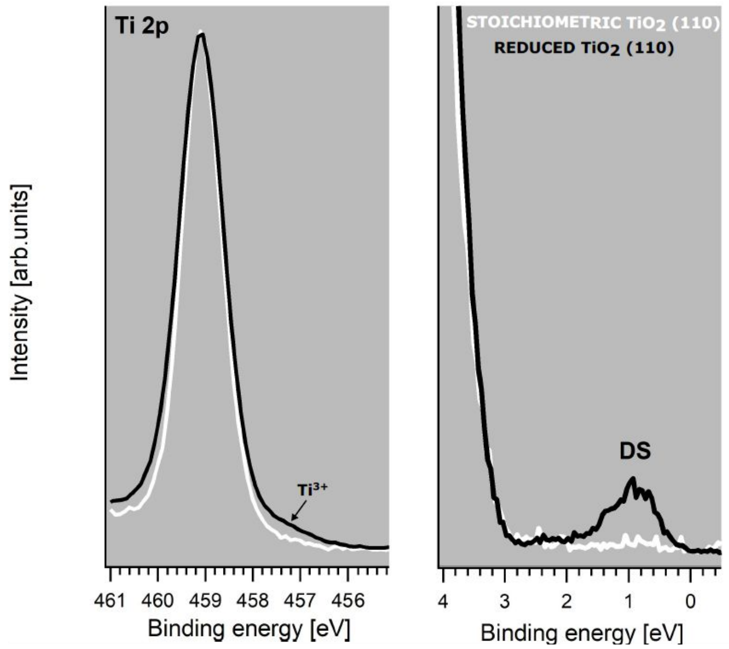

Figure S 1: Spectroscopic fingerprints of the stoichiometric (in white) and a reduced TiO_2 (110) (in black) surfaces. Ti2*p* CL (left) and the VB region (right) are shown. In this work we exclusively used samples presenting the white line shape.

LEED evolution of the DBTP structure upon Co deposition and subsequent annealing steps

Figure S2 shows the LEED evolution during the Ullmann coupling reaction of the cobalt sample (panels c-e). Panels a, b serves as a comparison where the pristine TiO_2(110) and a full monolayer of DBTP on TiO_2(110) are shown respectively. For the cobalt sample (panels c-e) a clear evolution of the LEED pattern becomes evident: at room temperature, the organometallic (C-Co-C) chains appear as vertical diffraction lines (panel c, highlighted with green arrows), after annealing to 420K, the characteristic diffraction lines of PPP polymers show up (panel d, highlighted with red arrows) and such PPP lines persist even after 600K annealing step (panel e).

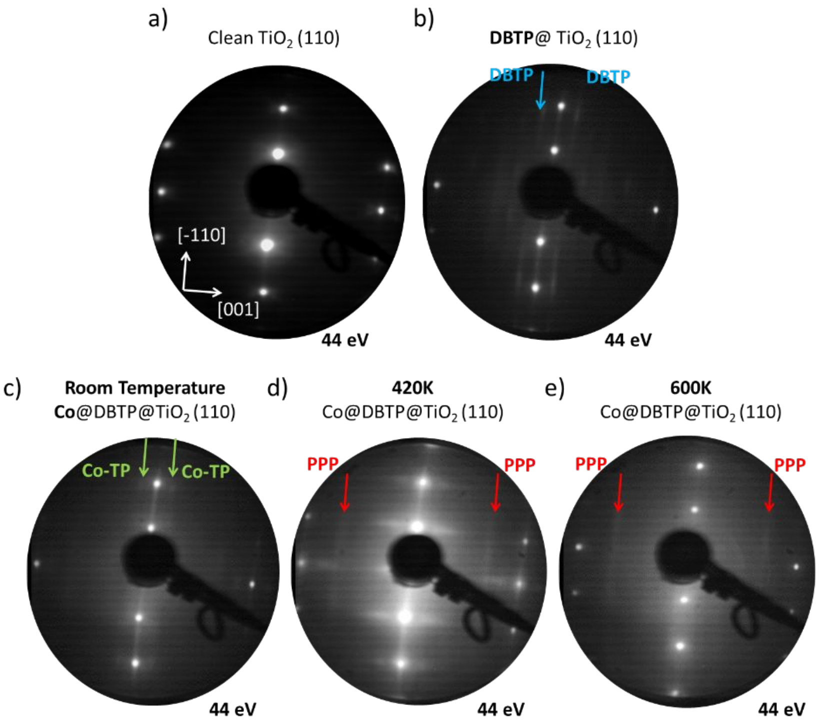

Figure S 2: a,b) LEED images taken at 44 eV corresponding to the non-reduced TiO_2 (110) pristine sample and one monolayer DBTP deposition on the surface. The new diffraction pattern arising from the DBTP molecules is highlighted with blue arrows. c-e) LEED images taken at 44 eV corresponding to the Ullmann coupling reaction of the cobalt sample. At room temperature the diffraction vertical lines corresponding to the organometallic phase (denoted as Co-TP) are observed (panel c). After 420 K annealing step (panel d) new diffraction lines (highlighted with red arrows) show up and arise from the periodicity of the PPP chains. Such PPP chain diffraction patterns persist even after annealing to 600 K (panel e) in agreement with the TD-XPS observations of Figure 2 in the main manuscript. Note that the LEED images are taken at RT after each annealing step.

<u>STM image of DBTP/Co/TiO_2(110) and DBTP/TiO_2(110) after polymerization</u>

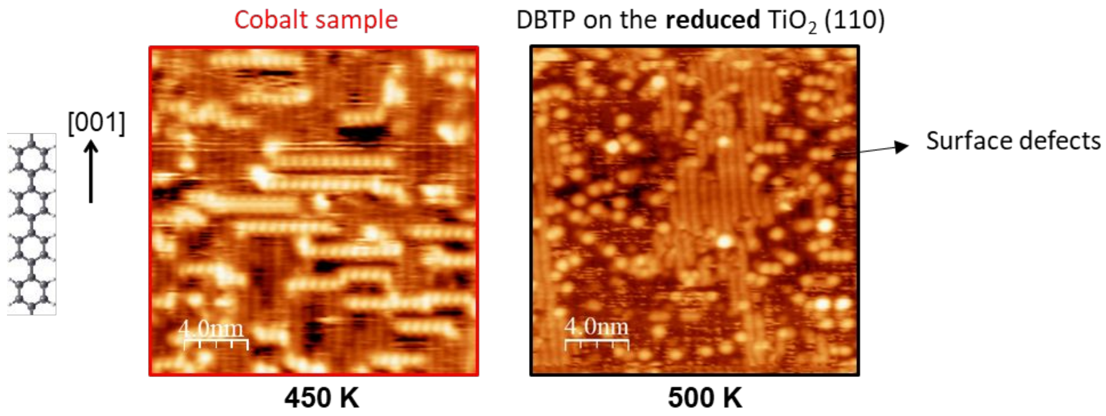

Figure S 3: RT-STM images of the cobalt sample, left, measured after the 450 K annealing step and the control sample measured on the reduced TiO_2(110) surface after the 500K annealing step (STM parameters: V= 1,8V, I= 35pA).

The limited surface conductivity of TiO_2(110), restricts the performance of STM as a suitable characterization technique for the current experiment. As a consequence, obtaining accurate images is highly challenging and the obtained results scarce.

The success of using cobalt atoms as catalysts for the Ullmann coupling reaction on TiO_2(110) has been proven by XPS, LEED and ARPES measurements. To gain insight into the formation of the Br-Co complex during the intermediate stages of the reaction, RT-STM was used after annealing the cobalt sample to 450K. In the STM image shown on the left in Figure S3, two distinct structures are resolved perpendicular to each other. On the one hand, with lower intensity, PPP chains are present on the surface along the [001] high-surface anisotropy direction. The polymerization rate and polymer length can not be determined from the images at this stage.

A different, higher intensity structure perpendicular to the [001] direction also appears in the image. This structure is not observed after the polymerization reaction in the absence of cobalt atoms, as shown in the STM Figure S3 right panel, and has not been reported in previous similar works. It can neither be attributed to the characteristic TiO_2(110) 2x1 surface reconstruction, as evidenced by the absence of a surface defect state peak in the valence band spectra[4] (SI Figure S1). Therefore, we correlate the observed structure with the stable Br-Co complex.

For comparison purposes, in the right panel of the Figure S3, we include an STM image of PPP grown on the reduced TiO_2(110) sample after annealing a DBTP monolayer up to 500K. Due to the low reaction yield, the surface is only partially covered by PPP polymers. The bright point like protrusions correspond to the typical surface defects of the reduced TiO2(110),

extensively studied and characterized so far and attributed to surface oxygen vacancies and hydroxyl groups.

<u>ARPES spectra comparison of DBTP/TiO_2(110) and DBTP/Co/TiO_2(110) systems at RT and at polymerization temperature</u>

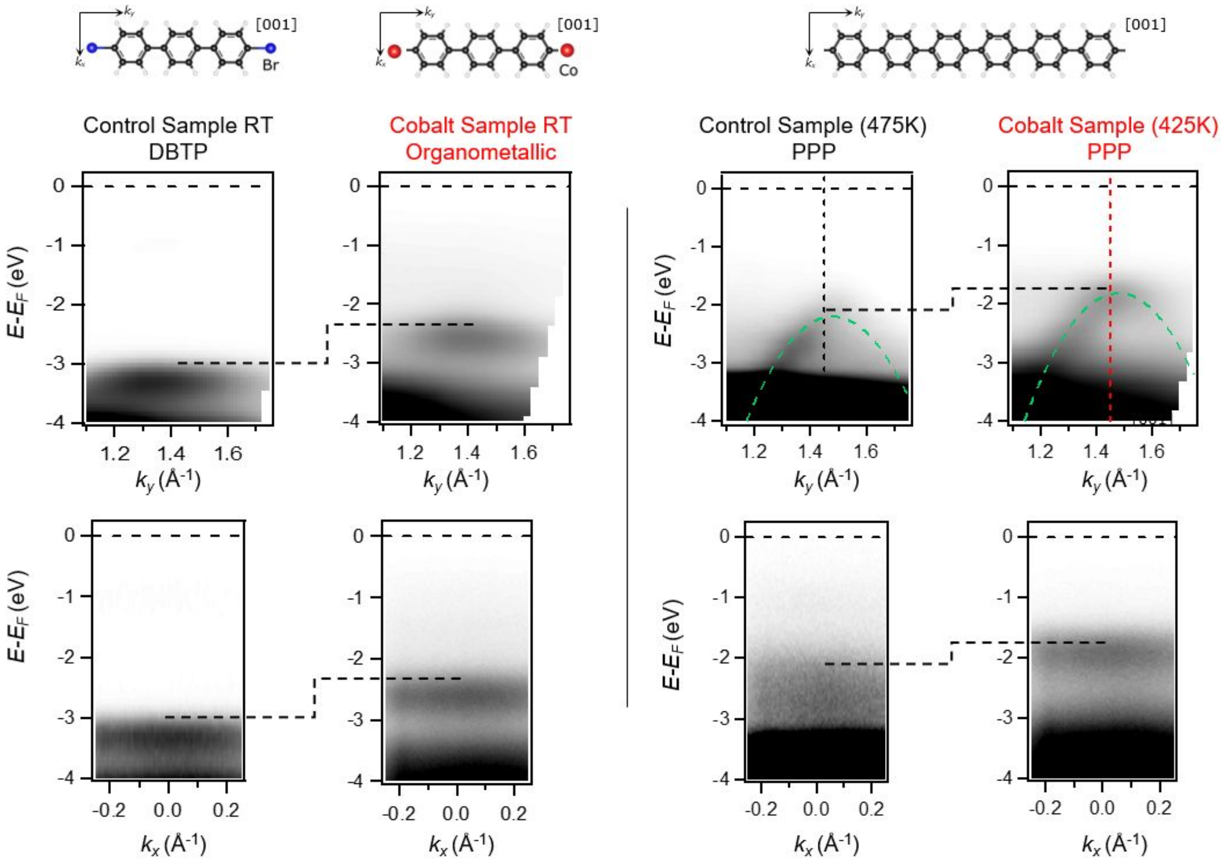

Figure S4: Band structure measured by ARPES for both control and cobalt samples on TiO2(110) at RT (a) and after annealing to 475K and 425K, respectively (b). In (a) (top), E vs k_y photoemission intensity maps are shown where the non-dispersive molecular orbital along the DBTP molecule axis is detected for both systems. At the bottom, E vs k_x photoemission intensity mapping is extracted at $k_y = 1.45$ Å^{-1}. In (b) (middle), E vs k_y photoemission intensity maps of PPP along the polymer axis are shown, and at the bottom, E vs k_x photoemission intensity mapping is extracted at $k_y = 1.45$ Å^{-1} for both systems. The chemical structure of DBTP, the organometallic intermediate, and PPP are depicted on top of the image.

The band structure of DBTP measured along its main molecular axis reveals a non-dispersive molecular band in both the control and the organometallic sample (Figure S4a, middle). Interestingly, the band shows a 0.6eV upward shift in the organometallic phase, induced by molecular dehalogenation and molecular interaction with the cobalt atoms. The preservation of the non-dispersive character of the bands, however, indicates that polymerization has not yet

occurred. The energy shift of the band is clearly depicted in the E vs k_x photoemission intensity mapping extracted at $k_y = 1.45$ Å^{-1} for both systems in Figure S4a, bottom.

Figure S4 b has already been described in the main text and serves as a clear evidence for the DBTP polymerization into PPP by cobalt atoms, as well as showing cobalt induced shift of the PPP band.

ARPES spectra of the DBTP/Co/TiO_2(110) system at different temperatures

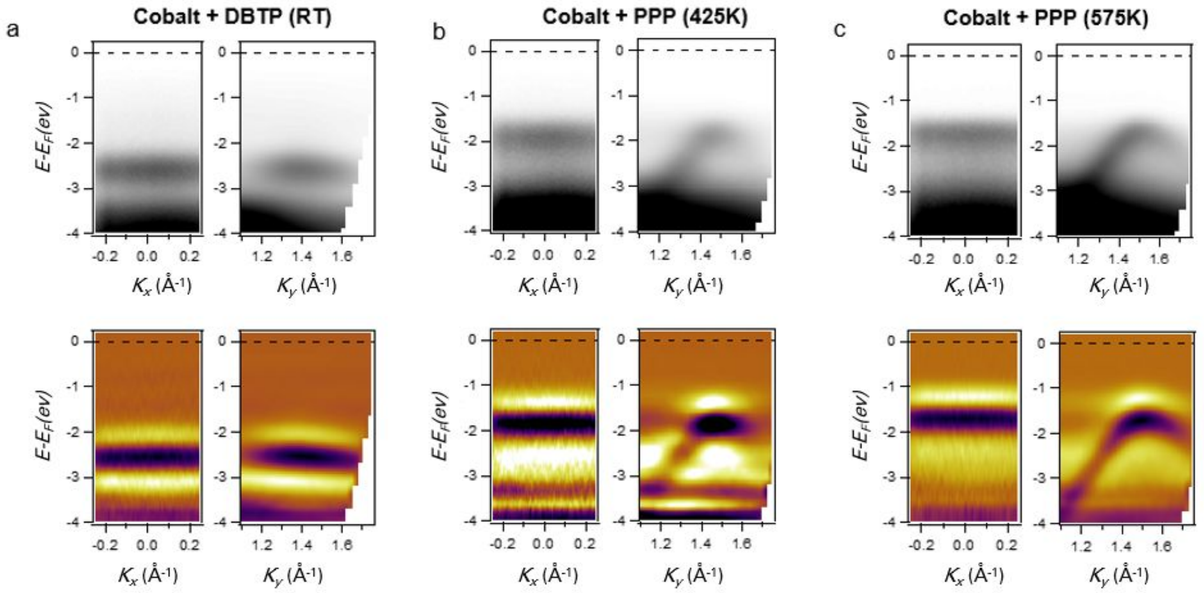

Figure S5: Photoemission intensity maps measured for DBTP/Co/TiO2(110) system at RT (a), 425K (b) and 575K (c). All E vs k_y intensity maps are measured along the main molecular and polymer axis's while E vs k_x maps are extracted at $k_y = 1.45$ Å^{-1}. The second derivative of the spectra are plotted bellow.

For visualization purposes, in **Figure S5**, the second derivative ARPES spectra of the cobalt system are included in conjunction with the original data.

TD-XPS of DBTP and DITP with Co deposition

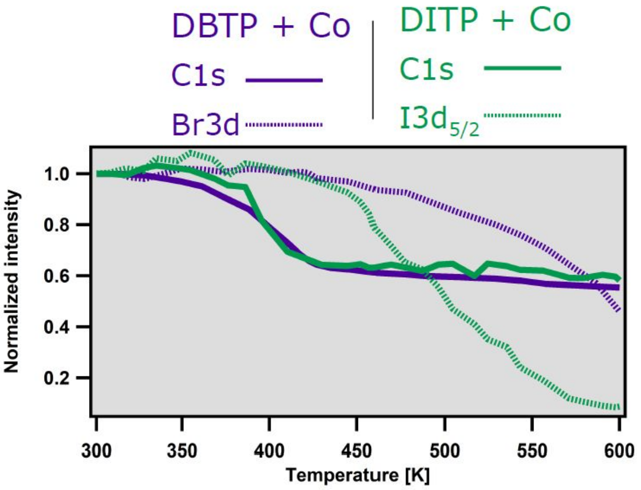

Figure S 6: TD-XPS comparison of the normalized intensity areas of the main core level features of DBTP and DITP in the cobalt samples.

Finally, for comparison purposes, we superimpose the normalized intensity spectra measured in Figure 2c and Figure 4c of the main manuscript in Figure S4. The normalized intensity spectra of the C1*s* CL in both molecular precursors, DBTP and DITP, have similar evolution and drop to 60% at 600K, indicating that the Ullmann coupling reaction is practically identical with both precursor molecules after dehalogenation. However, in contrast to the I3*d* signal trend, the Br3*d* signal gradually desorbs and is still present at 600K, likely due to a stronger chemical interaction between cobalt and bromine that prevents bromine from desorbing.

REFERENCES

(1) Horcas, I.; Fernández, R.; Gómez-Rodríguez, J. M.; Colchero, J.; Gómez-Herrero, J.; Baro, A. M. WSXM : A Software for Scanning Probe Microscopy and a Tool for Nanotechnology. Review of Scientific Instruments 2007, 78 (1), 013705. https://doi.org/10.1063/1.2432410.

(2) Momma, K.; Izumi, F. VESTA 3 for Three-Dimensional Visualization of Crystal, Volumetric and Morphology Data. J Appl Crystallogr 2011, 44 (6), 1272–1276. https://doi.org/10.1107/S0021889811038970.

(3) Krüger, P.; Bourgeois, S.; Domenichini, B.; Magnan, H.; Chandesris, D.; Le Fèvre, P.; Flank, A. M.; Jupille, J.; Floreano, L.; Cossaro, A.; Verdini, A.; Morgante, A. Defect States at the TiO 2 (110) Surface Probed by Resonant Photoelectron Diffraction. Phys. Rev. Lett. 2008, 100 (5), 055501. https://doi.org/10.1103/PhysRevLett.100.055501.

(4) Sánchez-Sánchez, C.; Garnier, M. G.; Aebi, P.; Blanco-Rey, M.; de Andres, P. L.; Martín-Gago, J. A.; López, M. F. Valence Band Electronic Structure Characterization of the Rutile TiO2 (110)-(1×2) Reconstructed Surface. Surface Science 2013, 608, 92–96. https://doi.org/10.1016/j.susc.2012.09.019.
